# Deep learning approach based on a patch residual for pediatric supracondylar subtle fracture detection

**DOI:** 10.17305/bb.2024.11341

**Published:** 2025-01-16

**Authors:** Qingming Ye, Zhilu Wang, Yi Lou, Yang Yang, Jue Hou, Zheng Liu, Weiguang Liu, Jiayu Li

**Affiliations:** 1 Zhejiang Sci-Tech University, Hangzhou, China; 2 Hangzhou Children’s Hospital, Hangzhou, China; 3 First Teaching Hospital of Tianjin University of Traditional Chinese Medicine, Tianjin, China

**Keywords:** Deep learning, DL, pediatric supracondylar subtle fracture, fracture detection, small data

## Abstract

Supracondylar humerus fractures in children are among the most common elbow fractures in pediatrics. However, their diagnosis can be particularly challenging due to the anatomical characteristics and imaging features of the pediatric skeleton. In recent years, convolutional neural networks (CNNs) have achieved notable success in medical image analysis, though their performance typically relies on large-scale, high-quality labeled datasets. Unfortunately, labeled samples for pediatric supracondylar fractures are scarce and difficult to obtain. To address this issue, this paper introduces a deep learning-based multi-scale patch residual network (MPR) for the automatic detection and localization of subtle pediatric supracondylar fractures. The MPR framework combines a CNN for automatic feature extraction with a multi-scale generative adversarial network to model skeletal integrity using healthy samples. By leveraging healthy images to learn the normal skeletal distribution, the approach reduces the dependency on labeled fracture data and effectively addresses the challenges posed by limited pediatric datasets. Datasets from two different hospitals were used, with data augmentation techniques applied during both training and validation. On an independent test set, the proposed model achieves an accuracy of 90.5%, with 89% sensitivity, 92% specificity, and an F1 score of 0.906—outperforming the diagnostic accuracy of emergency medicine physicians and approaching that of pediatric radiologists. Furthermore, the model demonstrates a fast inference speed of 1.1 s per sheet, underscoring its substantial potential for clinical application.

## Introduction

Supracondylar humerus fractures are the most common type of elbow fracture in children, but they pose distinct diagnostic challenges [1]. During skeletal growth, the ligaments surrounding a child’s elbow are more injury-prone than in adults due to the ongoing remodeling of the distal humerus and the thinning of the bone cortex. Minor or occult fractures may show minimal displacement or lack visible fracture lines, while the presence of ossification centers further complicates fracture identification on pediatric radiographs [2]. Detecting these fractures is particularly difficult for non-pediatric emergency physicians, as subtle fractures often lack clear markers like significant displacement or distinct fracture lines [3]. With rapid advancements in machine learning (ML) techniques, traditional bone detection methods have gained attention. These approaches typically rely on classical ML algorithms, such as support vector machines (SVMs) [4], random forests (RFs) [5], and hand-crafted feature extraction [6]. However, their dependence on domain-specific knowledge for feature design, coupled with limited generalization capabilities, makes it challenging to adapt them to the wide variability of anatomical structures. To overcome these limitations, convolutional neural networks (CNNs) have been introduced for bone detection due to their ability to automatically extract features [7–9]. Some methods combine feature extraction modules with classifiers to analyze radiological images and localize abnormal regions using visualization techniques like Grad-CAM [10]. However, these approaches often lack the specificity needed to detect fractures in children. While recent studies have successfully localized fractures in the hip [11], leg [12], and radial bones [13], they remain insufficient for identifying pediatric supracondylar humerus fractures. In recent years, DL has significantly advanced medical imaging, with CNNs achieving high diagnostic accuracy in radiology [14]. However, CNN models require large volumes of labeled data to perform effectively, and obtaining such data in medical contexts poses unique challenges. Labeling fracture images demands specialized physicians who can identify and annotate subtle features, such as faint fracture lines or irregularities in the bone cortex. This process is time-consuming, labor-intensive, and subject to variability among annotators, making it difficult to create reliable, large-scale datasets. These challenges are especially pronounced for supracondylar humerus fractures in children. The pediatric skeleton’s unique anatomical and developmental characteristics, including ossification centers and ongoing bone remodeling, obscure fracture features and complicate manual annotation. Additionally, the limited availability of pediatric supracondylar fracture images exacerbates the lack of labeled datasets [15]. Consequently, CNN-based models trained on small datasets struggle to generalize effectively to external datasets or unseen cases, such as images from different healthcare providers or imaging devices.

The abundance and easy access to healthy bone images provide new opportunities for advancing fracture detection. Medical experts often rely on their knowledge of normal anatomical structures to identify deviations indicative of fractures [16]. Inspired by this diagnostic approach, leveraging healthy images to learn the feature distribution of normal bones offers a promising solution to address the issue of data scarcity. Building on this concept, this paper introduces a DL-based multi-scale patch residual fracture detection network (MPR) designed to detect and localize subtle supracondylar fractures in children. The proposed framework utilizes healthy images to learn skeletal features and reconstruct fracture regions without requiring detailed fracture annotations. The MPR network incorporates a multi-scale generative adversarial network (MGAN) to capture bone-consistent features, combined with a weighted binary cross-entropy (W-BCE) loss function to improve the detection accuracy of subtle fractures. This method effectively leverages existing healthy data while reducing dependence on scarce annotated datasets. Using a generative adversarial network (GAN)-CNN hybrid architecture, the network emphasizes subtle distinctions between positive and negative samples, enhancing the representation of fracture characteristics in pediatric cases. By facilitating the calculation of residuals in fracture samples, the proposed model achieves precise and reliable detection. This also mitigates the negative impact of pediatric anatomical variability on the model’s performance, resulting in more accurate fracture identification. The primary contributions of this study are as follows.

*Automatic diagnosis model*: An automatic model for detecting subtle supracondylar fractures based on CNNs is proposed. It comprises three main components: elbow localization, subtle fracture repair, and fracture detection.

*Multi-scale architecture*: A multi-scale model built on the ConvNeXt backbone, paired with a non-rectangular bounding box localization method, is introduced to enhance fracture localization accuracy.

*High performance metrics*: The proposed model significantly improves detection performance, achieving 90.5% accuracy, 89% sensitivity, 92% specificity, and an F1 score of 0.906 on an independent test set. It surpasses emergency medicine physicians in accuracy and approaches the performance of pediatric radiologists. Additionally, it demonstrates excellent inference speed (1.1 s per image), highlighting its potential for clinical deployment. The remainder of this paper is structured as follows: Section II reviews related studies on GAN-based fracture detection and anomaly detection, and provides a detailed description of the proposed MPR framework. Section III presents and discusses the experiments and results. Finally, Section IV provides the conclusion and future perspectives of this study. This revision improves clarity, eliminates redundancy, and enhances readability while maintaining all key information.

## Materials and methods

### Fracture detection

Fracture detection is a vital aspect of clinical diagnostics, particularly in radiology, where image processing technologies are increasingly used to assist physicians in identifying fractures. Methods for fracture detection typically fall into two categories: conventional ML methods and DL methods, each with its own strengths and limitations. The choice between them depends on the specific application context. Conventional ML methods rely on manually extracted features from radiographic images, which are then input into classifiers to predict the presence and type of fractures. The effectiveness of these methods depends heavily on the quality of selected features and classifiers. For example: Umadevi et al. [17] extracted texture and shape features from tibial images and tested three classifiers: back propagation neural networks, K-nearest neighbors (KNN), and SVM. They found that ensemble models, which combine multiple classifiers, significantly improved fracture recognition quality. Lum et al. [18] compared various classifier combinations (e.g., Bayesian and SVM) for femoral and radial fracture detection, achieving excellent results. Al-Ayyoub et al. [19] employed four classifiers—decision trees (DT), SVM, naive Bayes, and neural networks—for long bone fracture classification. SVM achieved the best accuracy, exceeding 85% after 10-fold cross-validation. Other researchers have explored innovative feature extraction techniques to enhance performance. For instance: Cao et al. [20] introduced a stacked RF feature fusion approach that combined multiple feature types, improving fracture detection accuracy. Scholars in [21] extracted Harris corner points from sharpened tibial images and applied DT and KNN for fracture detection and classification. However, these methods are not without limitations. Manual feature extraction is time-consuming, requires domain expertise, and can introduce subjectivity. Additionally, conventional ML models often struggle to generalize to new fracture types or radiological conditions due to incomplete feature sets. In contrast, DL—particularly CNNs—has transformed fracture detection by eliminating the need for manual feature extraction. CNNs automatically learn image features, such as shapes, edges, and textures, to make predictions. This capability has enabled CNNs to excel in diverse fracture detection scenarios [22]. For example: Hendrix et al. [23] developed a CNN model for scaphoid fracture detection in wrist and hand images, achieving performance comparable to radiologists. Other studies [24, 25] demonstrated CNNs’ effectiveness in pediatric elbow fracture detection and radial/ulnar fracture localization in wrist X-rays. Guan et al. [26] proposed an improved CNN model for arm bone X-ray fracture detection, achieving state-of-the-art average precision (AP). Recent studies have further advanced CNN applications: Researchers [27] fine-tuned pre-trained models (e.g., AlexNet and GoogleNet) on 2009 X-ray images, achieving 99.56% accuracy. Saliency maps were incorporated to enhance interpretability. However, reliance on small datasets and pre-trained models limited generalizability. A DL approach [28, 29] using oversampling and transfer learning with AlexNet and ResNet50 achieved higher accuracy than traditional methods but faced challenges with small datasets. By combining redefined residual learning with CNNs, researchers improved performance on complex skeletal lesions but faced limited generalization on larger datasets. An interpretable DL model [30] using transfer learning (ResNet, AlexNet) achieved 95% accuracy. Saliency maps and feature visualization enhanced clinical transparency but highlighted challenges with imaging variability and patient diversity. Despite their promise, DL methods face common challenges. Small dataset sizes often limit model training and generalizability. Variability in imaging protocols and patient populations further complicates real-world deployment. Nevertheless, advancements in DL continue to drive improvements in fracture detection accuracy and clinical decision-making transparency.

In addition to CNN-based networks, transformer-based approaches are increasingly being adopted for fracture detection. For example, Tanzi et al. [31] highlight the use of Vision Transformers (ViT) for classifying femur fractures, demonstrating that ViT outperforms traditional CNNs and enhances the accuracy of specialist diagnoses. Similarly, Sarmadi et al. [32] explore the application of ViTs for medical image analysis, specifically in diagnosing osteoporosis from X-ray images. Their study concludes that ViTs outperform CNNs when sufficient training data is available. Nejad et al. [33] address the limited research on DL techniques for cervical spine fracture detection. They propose a two-stage pipeline that integrates a Global Context ViT for vertebrae detection with a YOLOv8 model for fracture detection. Their approach outperforms popular models such as YOLOv5 in terms of effectiveness. Zhu et al. [34] introduce a novel cross-view deformable transformer framework for detecting non-displaced hip fractures. Their method overcomes challenges in identifying discriminative features and extracting complementary information from paired frontal and lateral X-ray images. This is achieved through a deformable self-attention module and cross-view representation modeling, which they evaluate on a dataset of 768 hip cases. Liu et al. [35] propose a hybrid transformer-CNN (HTCNN)-based radiomics model for osteoporosis screening using routine CT scans. Their model achieves superior segmentation precision and better osteoporosis discrimination compared to traditional Hounsfield unit (HU) values, boasting high AUC scores in both training and test cohorts. Additionally, Makwane et al. [36] present a hybrid approach that combines the Swin transformer with traditional feature extraction methods like SIFT for fracture identification and classification. Their extensive experiments demonstrate the hybrid model’s effectiveness in improving clinical decision-making for fracture diagnosis. Despite the advantages of ViTs in fracture detection, they are not without challenges. One notable drawback is their slower speed and longer training times compared to traditional CNNs, primarily due to the computational complexity of the self-attention mechanism. Furthermore, ViTs generally require large amounts of labeled data to perform well, limiting their applicability to tasks with smaller datasets. These constraints make ViTs less efficient for real-time applications and less suitable for scenarios with limited annotated medical images. Consequently, we chose CNN-based models for our fracture detection task. While DL models, including CNNs and pre-trained architectures, have significantly advanced fracture detection, they still face obstacles, such as data scarcity and limited generalization. These studies underscore that, although pre-trained models and transfer learning techniques improve accuracy, their performance often hinges on the size and diversity of the datasets. Even with high accuracy on specific datasets, these models struggle to generalize across different patient populations and imaging conditions, posing a substantial challenge for their clinical application.

### Abnormal detection with GANs

With the growing challenge of data scarcity, anomaly detection methods based on GANs have emerged as a promising solution. By learning the distribution of normal data, GANs can reconstruct healthy data and identify deviations as anomalies, eliminating the need for labeled datasets. Numerous studies have explored the potential of GAN-based anomaly detection in medical image analysis [37, 38]. For example, Schlegl et al. [39] developed a GAN trained on healthy data and proposed a method to quickly map new inputs into the GAN’s latent space. Anomalies were identified using a comprehensive anomaly score derived from discriminative residual and reconstruction errors. Zhao et al. [40] introduced a reconstruction-based approach that learns the manifold of normal data through encoding-reconstruction transformations between image and latent spaces. Reconstructed features were then utilized to distinguish anomalies from healthy data. Similarly, Zhou et al. [41] proposed Sparse-GAN, a novel framework for disease screening trained exclusively on healthy data. This method identifies anomalies in the latent space using features constrained by a sparse regularization network. These studies highlight the efficacy of GANs in detecting anomalies that deviate from the normal data distribution, with strong performance demonstrated on publicly available medical datasets, such as OCT and chest X-ray images [42]. However, their application to fracture detection remains underexplored. This study leverages GANs for anomaly detection and integrates a MPR network to address the challenge of detecting subtle supracondylar fractures. Unlike traditional CNN-based models that require large-scale annotated datasets, this approach trains GANs to learn the distribution of normal bone structures, overcoming the limitation of limited labeled data. By incorporating multi-scale CNNs with residual information, the proposed model accurately localizes fracture regions and improves the detection of subtle fractures. In contrast to existing GAN-based applications, this method not only leverages normal data for enhanced feature learning but also integrates auxiliary information (e.g., texture and edges) to boost the accuracy of fracture region identification. Consequently, the proposed approach demonstrates superior performance in detecting subtle fractures.

### Methods

**Figure 1.**
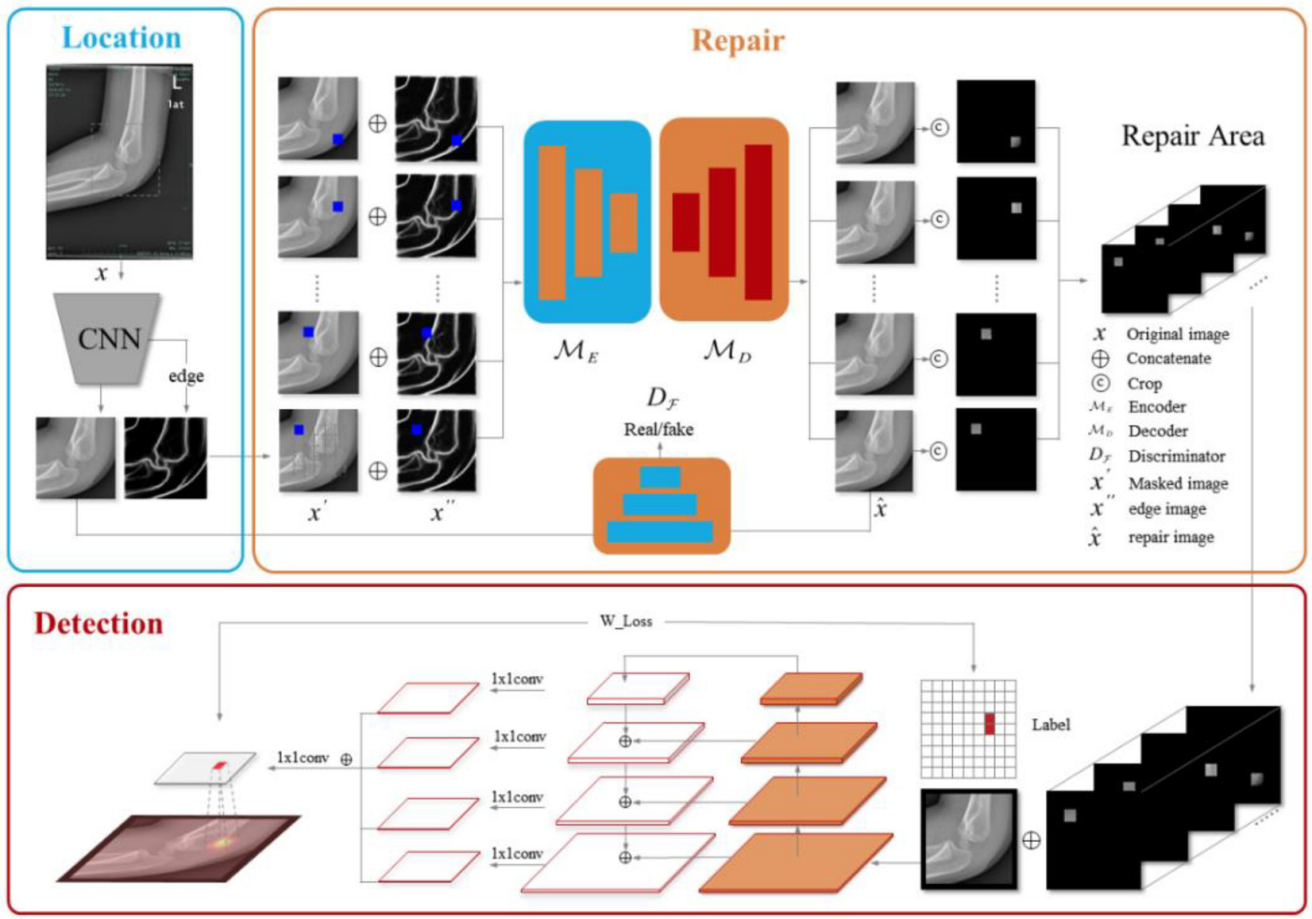
**Overall architecture of the proposed MPR approach.** The blue box shows the location model. The obscured area repair process is illustrated in the orange box. The fracture detection and location are presented in the red box. CNN: Convolutional neural network; MPR: Multi-scale patch residual network.

To overcome the limitations of traditional DL models in detecting subtle supracondylar fractures in pediatric elbow X-rays, we propose an MPR framework. This framework comprises three core modules: (1) an elbow localization module that focuses on the region of interest, (2) a skeletal region repair module that enhances fracture features through reconstruction, and (3) a fracture detection module that accurately localizes and classifies fractures. The overall architecture is illustrated in Figure 1.

### Elbow localization

The supracondylar region of the humerus in children is relatively small, occupying only a minor portion of X-ray images, which makes global analysis both inefficient and error-prone. To address this challenge, we utilized the YOLOv5 object detection model [43] to automatically localize the elbow region. YOLOv5 was selected due to its optimal balance between accuracy and real-time performance, making it particularly suitable for clinical applications. The localization module was trained to detect the elbow region and crop it into standardized 256×256-pixel images. This preprocessing step ensures that subsequent models concentrate solely on the target region while also reducing computational load. Importantly, the cropped images preserve essential anatomical details, providing a robust foundation for subsequent reconstruction and detection tasks.

### Skeletal region repair

**Figure 2.**
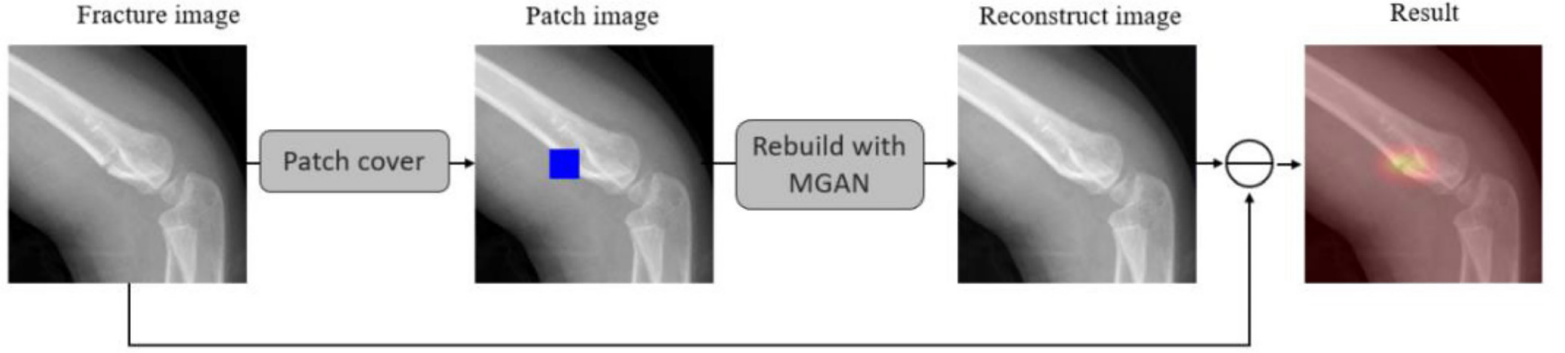
**Proposed scheme for residual detection between a fracture input image and a healthy rebuilt image.** MGAN: Multi-scale generative adversarial network.

In X-ray images, fracture textures are often identified as potential fracture regions. However, due to the subtle differences between positive and negative samples, previous methods have struggled to detect hairline fractures effectively. To address this challenge, we designed a marker specifically for healthy samples. As shown in Figure 2, this marker allows for the computation of residuals in fracture samples with greater ease. The reconstruction module is responsible for rebuilding damaged or occluded regions within cropped images. The output generated by this module emphasizes fracture features by highlighting the residual differences between the reconstructed image and the original. Notably, image reconstruction is a widely utilized task in computer vision.

**Figure 3.**
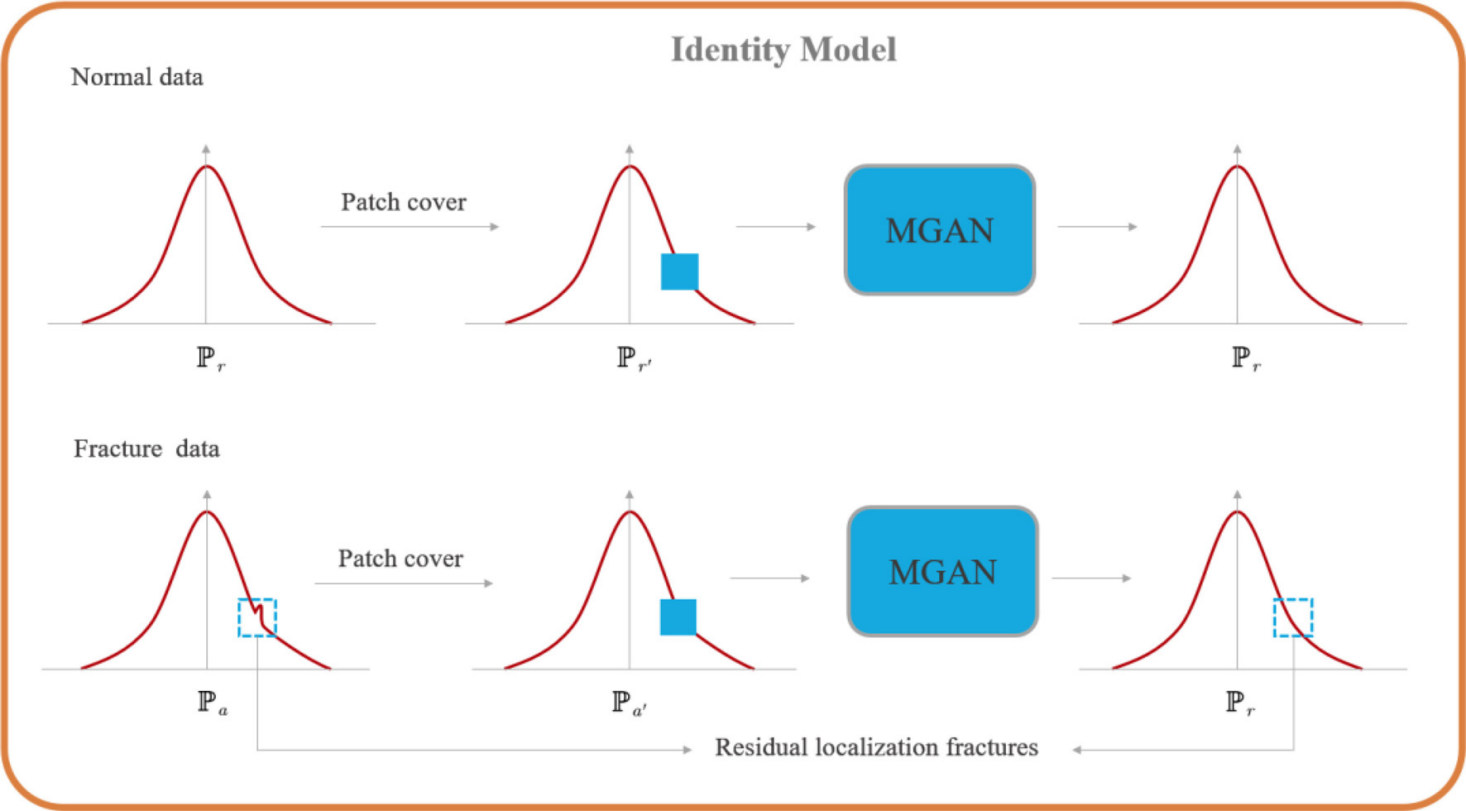
**Overview of the identity model for positive and negative samples.** MGAN: Multi-scale generative adversarial network.

Assuming we have a set of samples (i.e., *x*_i_ ═ {*x*_1_, *x*_2_,...,*x_i_*}), the reconstruction model generates corresponding X-ray images in a healthy state. This implies that the reconstructed results for healthy samples closely resemble their original counterparts, while the damaged regions in fracture samples are corrected. Thus, this process can be used to construct a recognition model: 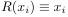.

To convert the reconstruction problem into a conditional generation problem, a small portion of the sample image is randomly occluded, and the remaining region is used as the condition. The generation problem for an unknown region in a healthy sample can be expressed as follows [44]: (1)
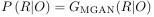
where *O* represents the occluded image region, and *R* denotes the reconstructed image region. As shown in Figure 3, by training the MGAN [38] using healthy normal data, the model learns to retrieve data from the distribution of healthy samples.

Intuitively, the difference between a healthy sample and a fracture sample can be clearly identified by a marker. The residual equation constructed based on this property is as follows [38]: (2)
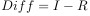
where *I* denotes the original image and *R* represents the generated image. The Diff obtained from the input model of healthy samples tends to zero, while the Diff obtained from abnormal samples presents a non-zero state.

**Figure 4.**
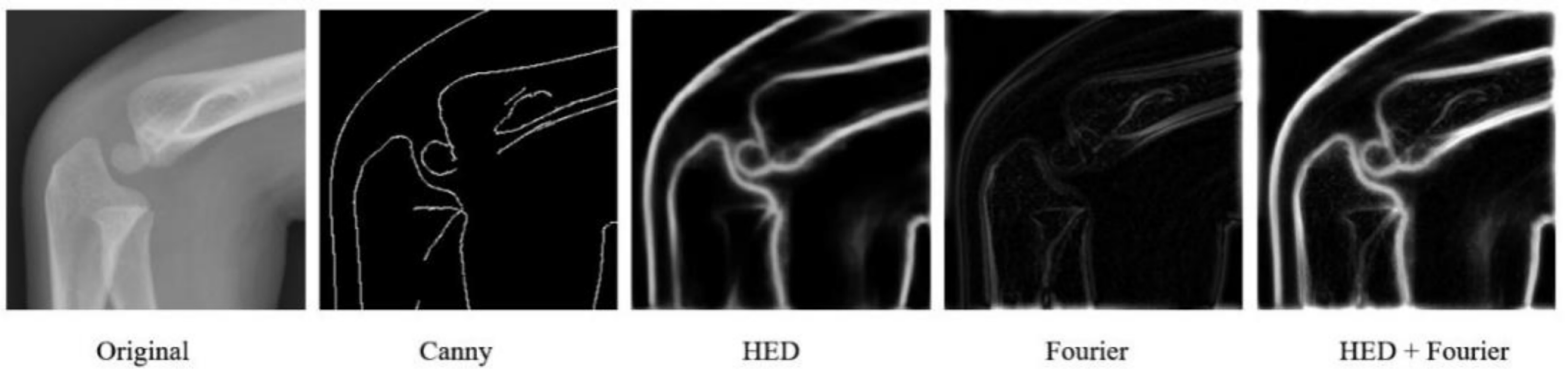
**Different methods used to obtain auxiliary information in the repair module.** From left to right, the original image, Canny, HED, Fourier, and HED + Fourier images are shown.

Edge information plays a critical role in reconstructing fracture regions. Fractures typically disrupt cortical bone and induce periosteal reactions, which often manifest as distinct edges in imaging. Without accurately capturing these edge details, reconstruction models may overlook crucial fracture-related information, leading to incorrect outcomes [45, 46]. To address this, we employed the HED network [47] to extract edge information in skeletal reconstruction. The HED network, based on deep CNNs, excels at capturing continuous edge details and is particularly well-suited for complex anatomical structures like pediatric elbow joints. Compared to traditional edge detection techniques, such as Canny [48], HED not only preserves precision but also provides more continuous and detailed edge information. This is vital for reconstructing fracture regions. Traditional methods like Canny are prone to noise sensitivity and struggle with intricate skeletal structures. These limitations are especially pronounced in pediatric bones, where ossification centers and unique growth patterns complicate the detection of subtle edge variations. In contrast, HED effectively addresses these challenges, delivering more accurate and detailed edge information, which helps the reconstruction model produce more realistic results (see Figure 4). In addition to edge information, texture characteristics of the fracture region are crucial during the repair process. Texture features enable the model to differentiate between normal bone and fracture regions, as fracture areas often exhibit distinct textural patterns compared to healthy bone. High-frequency texture variations frequently reveal minute fracture details, making them essential for the repair model. To capture these high-frequency texture features, we apply a 2D Fourier transform. This transform converts the image from the spatial domain to the frequency domain, highlighting high-frequency texture information. In fracture images—particularly those with microfractures or fine cracks—such features are represented by high-frequency variations. By leveraging the Fourier transform, we effectively detect these subtle changes and provide the repair model with detailed texture information, thereby enhancing the reconstruction process. The Fourier transform of the image is as follows: (3)
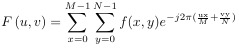

(4)
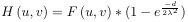


where the image size is (M, N), 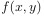 represents the pixel value of the image, and *d* represents the pixel centroid distance matrix with a constant λ of 10. Finally, 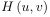 is the high frequency information in the frequency domain of the processed image. After applying an inverse Fourier transform to the data and converting it back to the spatial domain, the texture map shown in Figure 4 is generated. The impact of various auxiliary information on the restoration effect will be discussed in the following sections.

We define the bone region reconstruction task as follows: Given a large training dataset of expert-screened normal elbow anterior-posterior and lateral X-ray images, along with similar test images, the objective is to train a model to learn the distribution of healthy bone structures and reconstruct occluded regions to resemble these structures. To achieve this, we utilized the backbone network of the classical MGAN to construct both the generator and discriminator. In our method, an occluded image undergoes adversarial reconstruction, as illustrated in the orange box of Figure 1. Specifically, this process involves the supracondylar bone region in the cropped image x′x′x′ and an auxiliary image containing bone edge textures x
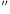
x
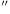
x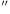. All cropped images are uniformly resized to 256 × 256 256 × 256 256 ×256 pixels. To ensure that the epicondylar skeletal region is adequately covered, the central masking area is set to 160 × 160 160 × 160 160 × 160 pixels. As shown in Figure 1, the gray dashed line divides the masking area into 25 regions, each of which is filled with a solid color mask. During training, the encoder (MEM_EME), decoder (MDM_DMD), and discriminator (DfD_fDf) are optimized alternately. The entire reconstruction model is supervised using a loss function that incorporates three components: adversarial loss, perceptual loss, and region reconstruction loss. These specific losses are defined as follows.

*Adversarial loss:* As shown in Figure 1, the objective of the discriminator is to distinguish between the original normal image and the image reconstructed by the generator. Through adversarial training with the discriminator, the encoder–decoder’s feature extraction and image reconstruction abilities are enhanced. Furthermore, the encoder and decoder progressively align with the distribution of real images, thereby “fooling” the discriminator. The adversarial loss that supervises the relationship between the encoder–decoder and the discriminator is expressed by the following formula [49]: (5)
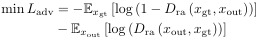
where 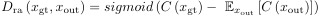 and the data pairs 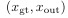 are sampled from the ground truth and output images.

*Perceptual loss:* In addition to the adversarial loss, which aligns the distribution of the synthetic image with the real image, we compute the perceptual error to capture high-level semantics and simulate human perception of image quality. We apply perceptual loss [50] in the encoder–decoder. Unlike the commonly used pixel-wise mean squared error (MSE) loss, perceptual loss focuses on high-level features and global information between the output image and the ground truth. The perceptual loss used to optimize the encoder–decoder is defined as follows: (6)
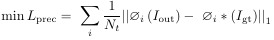
where 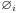 is the activation map of the *i*-th layer of the VGG-16 backbone.

**Figure 5.**
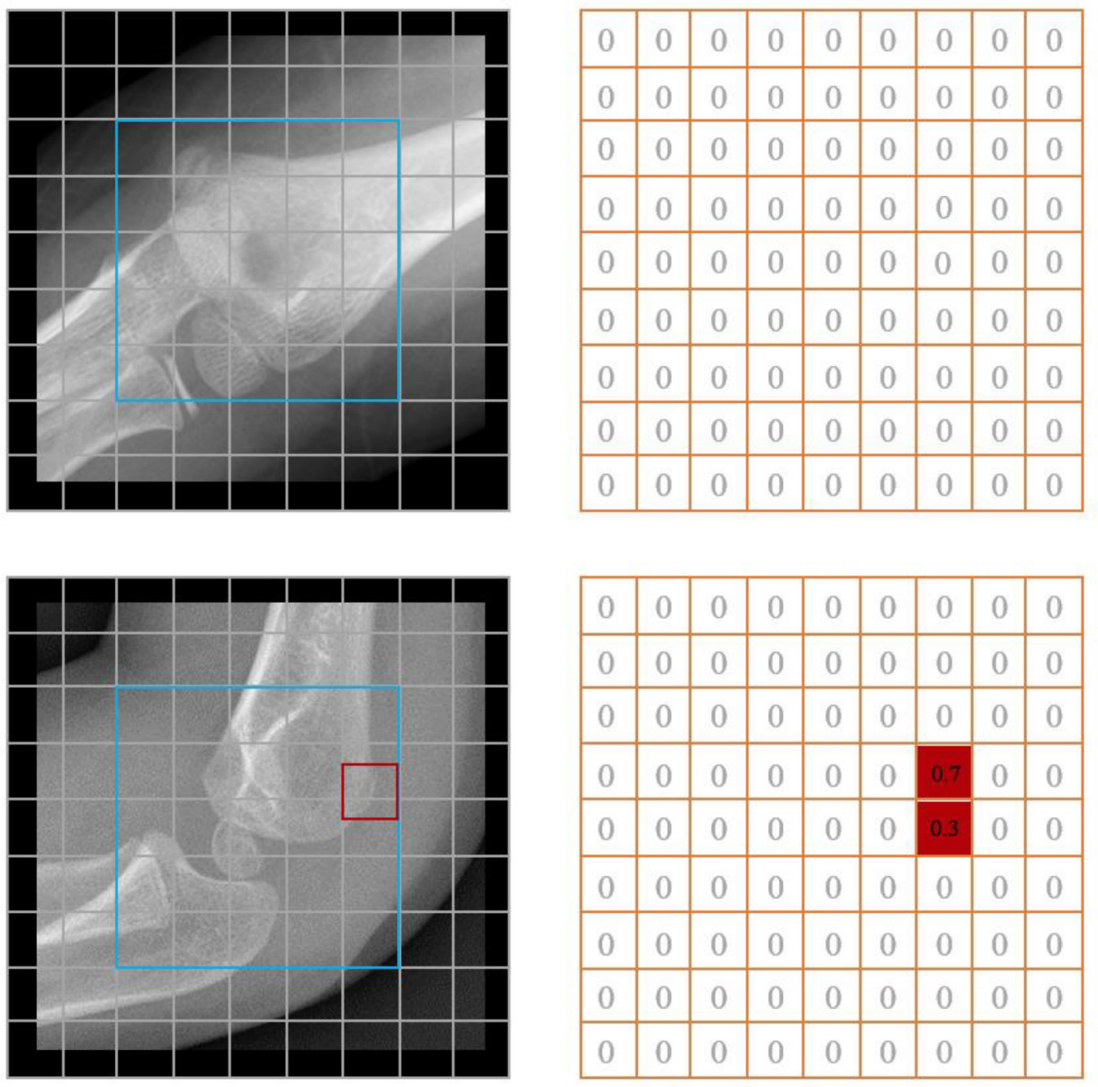
**The blue box indicates the repair area, while the red box represents the fracture annotation area.** The data label is a 9×9 two-dimensional array.

*Region reconstruction loss*: As noted previously, we consider both structural and semantic information in the image after reconstruction. Additionally, to enhance the model’s ability to repair fractures, we measure the pixel differences before and after the reconstruction of the occluded regions. Since the mask contains positional information, the repaired regions are accessible. We use pixel-level loss to guide this measurement task and improve the representation learning. This can be written as: (7)
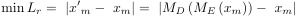
where 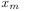 is the masked area image and 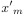 is the restored result.

Finally, the overall loss of the encoder 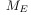, decoder 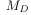, and discriminator 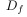 is optimized using the previously defined loss function as follows: (8)
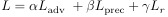
where 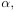 β, and γ are the loss weights.

### Fracture detection

The module is a critical component of the MPR framework, designed to identify and localize fracture regions within images. It combines multi-scale CNNs with FPNs to effectively extract multi-scale features, enabling the accurate detection and localization of subtle fractures, as illustrated in Figure 1. The primary objective of this module is to leverage repaired residual images alongside a high-efficiency DL model to precisely pinpoint fracture regions, particularly within complex and small fracture structures. To achieve this, we trained a multi-scale CNN on a labeled dataset containing both fracture and non-fracture images.

This dataset (i.e., 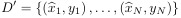) contains both fracture and non-fracture images, 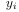 is a two-dimensional array where the value at each location corresponds to the area of overlap between the bounding box and the repaired region, as illustrated in Figure 5.

**Figure 6.**
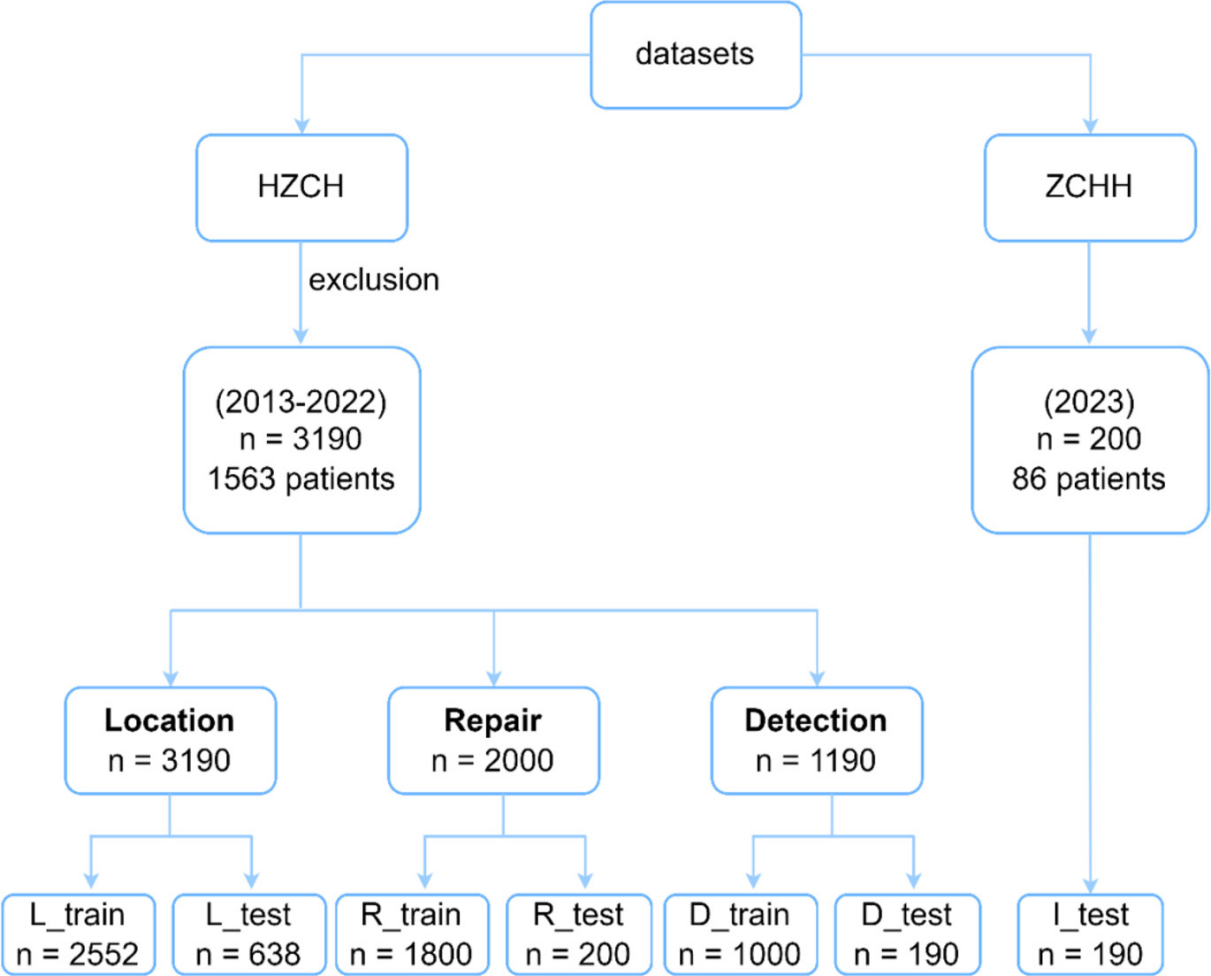
**Overview of the dataset**.

Additionally, we utilized ConnNeXt as the backbone feed-forward neural network and integrated it with a feature pyramid network (FPN) [51]. FPN, with its top-down feature fusion structure, extracts finer details from higher-level features while enriching lower-level features with this information. This design is especially important for fracture detection, as fracture regions often exhibit varying shapes and characteristics across different scales. Once the FPN generates feature maps at multiple spatial resolutions, these maps undergo downsampling and 1×1 convolutions to produce feature maps of the same size as the labels. The resulting feature maps are then concatenated and further convolved to generate the final output. During training, this output is compared with the supervised labels to compute the training loss. Specifically, we represent the image as a grid of cells, with each small block acting as a classification target. In typical fracture images, there is a significant class imbalance: 95% of the label array values are zeros, representing normal regions. Class imbalance is a common issue in fracture detection models, particularly for subtle fractures where fracture regions are significantly smaller than non-fracture regions. Using standard binary cross-entropy (BCE) loss in such cases may lead to overfitting on the non-fracture regions while neglecting fracture regions. To address this challenge, we employed a W-BCE loss [52]. The W-BCE loss function dynamically adjusts the importance of different classes, ensuring that fracture region features are not overlooked during training. By assigning higher weights to fracture regions, W-BCE encourages the model to focus more on this minority class, thereby improving sensitivity and detection accuracy. This approach is particularly advantageous for subtle fracture detection, as it enhances the model’s ability to identify fracture regions while mitigating the effects of class imbalance. The formula for the W-BCE loss is expressed as follows: (9)
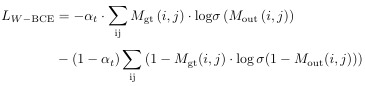
where 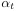 denotes the ratio of the nonfracture patches to total patches and σ(⋅) denotes the sigmoid activation function. *M*_out_(*i,j*) and *M*_gt_(*i,j*) represent the values under the output and label indexes, respectively.

### Ethical statement

Ethical approval for this study was obtained from the Institutional Review Board of Hangzhou Children’s Hospital (HZCH). All participants, including the guardians of pediatric patients, were provided with detailed information about the study and signed a written informed consent form. The study adhered to the Declaration of Helsinki and complied with all applicable ethical guidelines.

## Results

In this section, we evaluate the performance of our proposed MPR framework in detecting subtle supracondylar fractures in children, utilizing data from two medical institutions. We begin by describing the details of the datasets used in our study. Next, we present the experimental results for the localization, repair, and final detection models. Furthermore, we conduct ablation studies on the repair and detection models to assess the impact of edge and texture information on the repair process, as well as the role of the repair model in improving detection outcomes. Finally, we compare our experimental results with those achieved by existing methods.

### Datasets

An overview of the dataset is shown in Figure 6. All elbow X-ray images were obtained from the Picture Archiving and Communication System (PACS) at HZCH. These images were reviewed and annotated by multiple pediatric radiologists to reach a consensus. Images containing metal implants, displaced fractures, or positive fat pad signs were excluded. In total, 3190 X-ray images were collected from 1563 patients between 2013 and 2022, comprising 2585 healthy images and 585 fracture images. In this study, we adopted an image-based partitioning method rather than a patient-based partitioning approach. While this choice offers practical advantages, it could also introduce the risk of data leakage, as multiple similar images from the same patient might appear in both the training and testing sets. This overlap could potentially inflate the model’s performance, as the model might memorize patient-specific patterns instead of learning generalizable features [53–55]. To mitigate these potential risks, we implemented several strategies. To approximate the effect of patient-based partitioning, we applied advanced data augmentation techniques to increase the diversity of images from the same patient. These techniques included adding Gaussian or speckle noise to simulate variations in image quality, adjusting brightness and contrast to mimic different radiographic conditions, and applying slight translations, rotations, scaling, and flips to introduce positional variations. Additionally, elastic transformations were used to simulate soft tissue movement or minor anatomical structural changes. Furthermore, we incorporated regularization techniques, such as dropout and weight decay, during model training to reduce the likelihood of overfitting to patient-specific patterns.

Fractures, especially pediatric elbow fractures, are frequently underdiagnosed. To minimize annotation errors, we implemented a multi-expert review process. Three pediatric orthopedic specialists independently annotated the X-ray images. If at least two experts agreed on the presence of a fracture, the image was labeled accordingly. In cases where only one expert identified a fracture while the other two disagreed, the image was escalated for review by a senior specialist to provide the final annotation. The dataset consisted of 3190 X-ray images, which were used to train and evaluate various models. The dataset was randomly split into training and test sets. All 3190 images were used for training the localization model. From the 2585 normal images, 2000 were randomly selected for training and testing the reconstruction model. The remaining 1190 images were split into two subsets: D_train (1000 images) for training and D_test (190 images) for evaluating the detection model. To assess the model’s generalization ability, an I_test comprising 200 X-ray images from 86 cases was collected in 2023 at Zhongshan Hospital, affiliated with Huazhong University of Science and Technology. This independent test set was completely separate from the training and testing datasets and was used to ensure an unbiased evaluation of the model’s performance.

### Experimental

The proposed method was implemented using the PyTorch library. For the experiments, a 16GB Nvidia GeForce GTX 3080 Ti GPU was utilized for training. To standardize image sizes, bicubic interpolation was applied. The network was optimized using the Adaptive Moment Estimation (Adam) optimizer, with an initial learning rate of 0.0001 that decayed by 5% every 5 epochs. This learning rate schedule enabled larger updates during the initial stages of training and gradual fine-tuning as the model converged, ensuring stable training and improved generalization. To mitigate overfitting, dropout was applied with a rate of 0.5, randomly deactivating half of the neurons during each training iteration. This technique prevented the model from relying too heavily on specific features, encouraging it to learn more robust and generalized patterns. Additionally, early stopping was implemented to terminate training if the validation loss ceased improving, reducing the risk of overfitting and saving computational resources. Given the importance of execution speed in clinical applications, both training time and inference time were evaluated. The model required approximately 8 h to train for 100 epochs, with an average inference time of 1.1 s per image on the GPU. These considerations ensure the method is both efficient and practical for real-world use. The specific training parameters are shown in Table 1.

**Figure 7.**
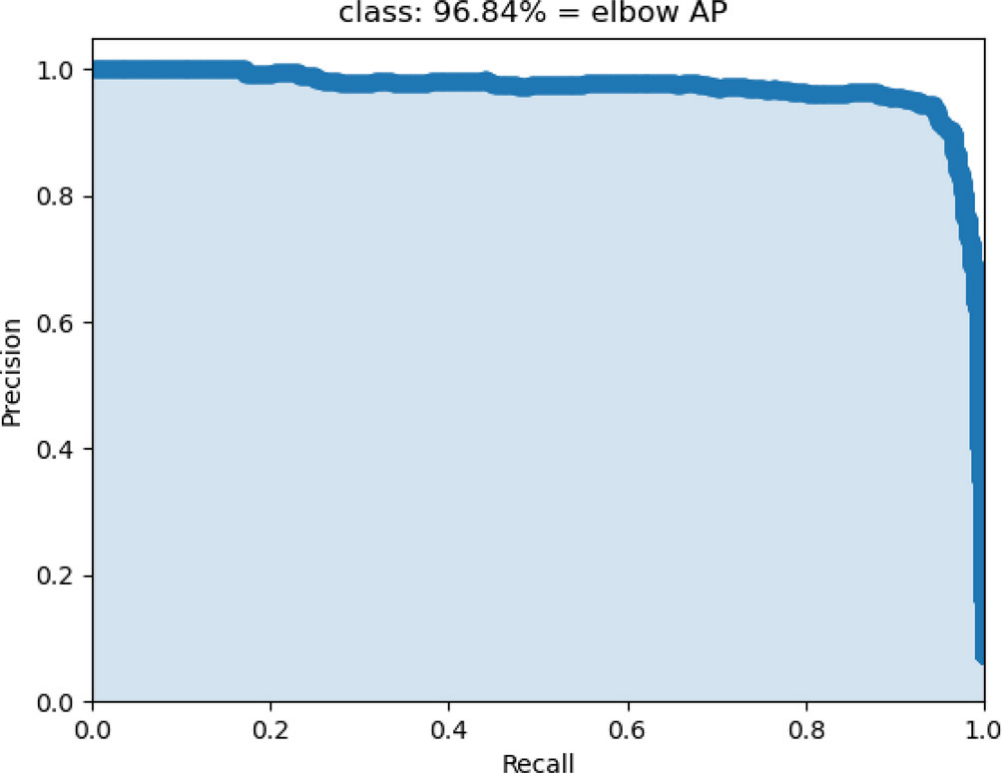
**Overview of the accuracy of elbow location.** AP: Average precision.

(1) *Results of the elbow localization model*: We initially evaluated the performance of our localization model using AP, a widely recognized metric for target detection. The YOLOv5 target detection model was utilized to localize the supracondylar bone in elbow radiographs. Figure 7 illustrates the experimental results on the test set, which demonstrate that the localization model achieved excellent accuracy. This outcome highlights the model’s ability to precisely identify the target. The high accuracy further indicates that the model can effectively differentiate the condyle from surrounding anatomical structures, thereby minimizing false positives. Nonetheless, it is important to note that the model’s success depends not only on its accuracy but also on its ability to generalize across diverse cases. Our test set included radiographs with varying levels of sharpness, ranging from high-resolution images with distinct bone landmarks to those with blurred bone structures and occlusions. The model’s ability to maintain strong performance under these challenging conditions underscores its robustness and adaptability to real-world clinical scenarios, where image quality may vary.

**Table 1:** Training hyperparameter values

**Name**	**Value**
Initial learn rate	0.0001
Max epochs	100
Batch size	8
Dropout	0.5
Learning drop factor	After 5 epochs if the accuracy has not increased
Optimizer	Adaptive moment estimation (Adam)

**Figure 8.**
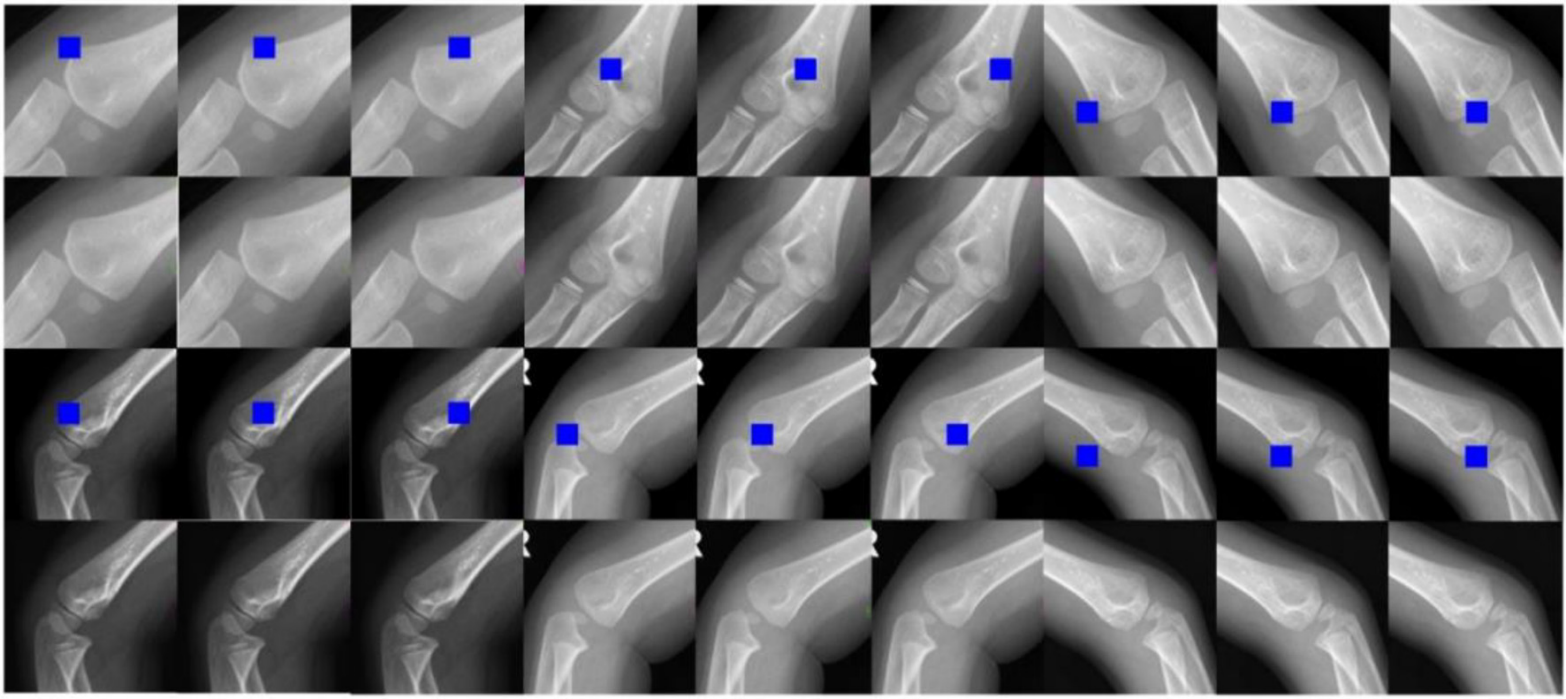
**Overview of the repair effect.** The nonfracture occlusion images and fracture occlusion images are shown from top to bottom.

**Figure 9.**
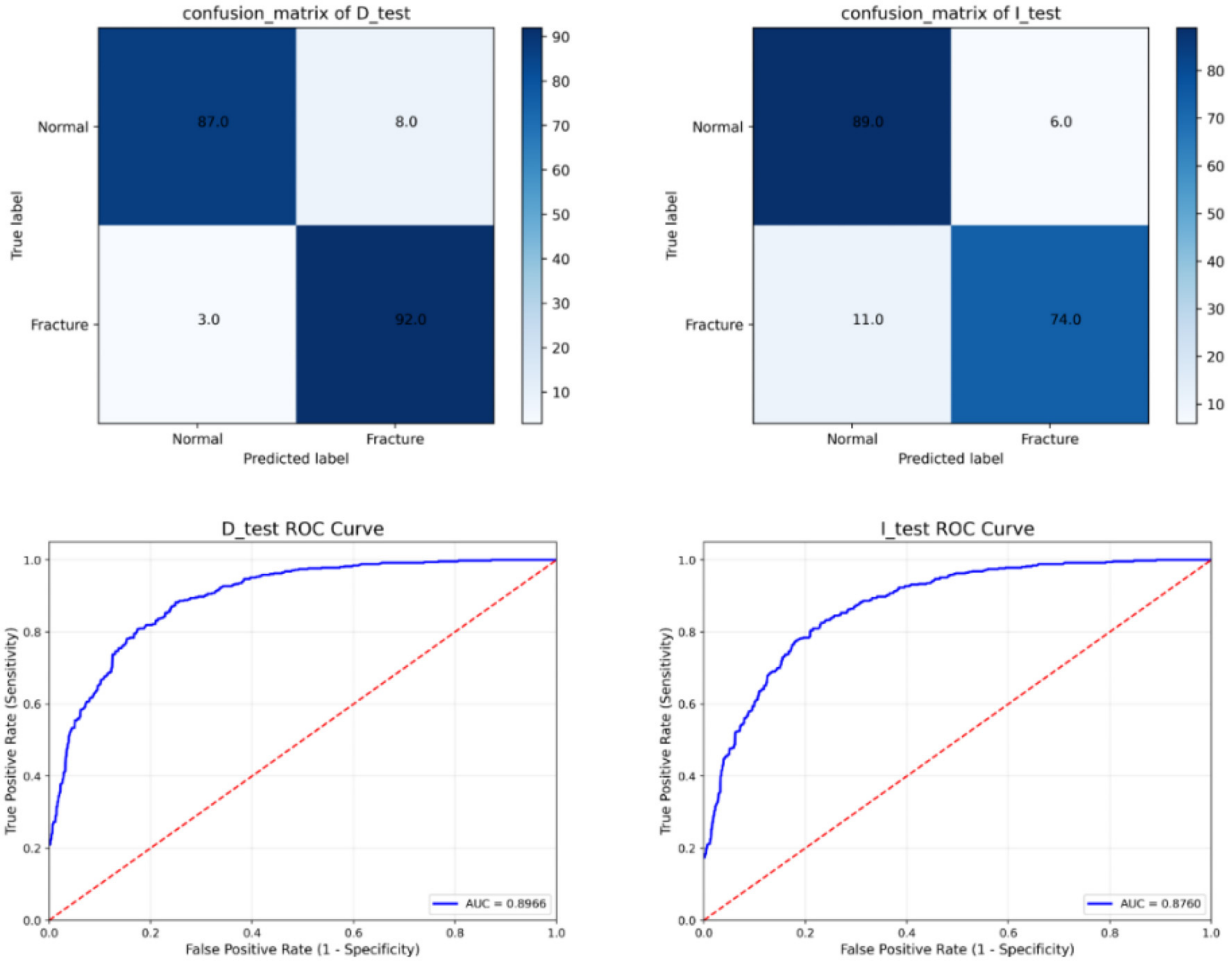
**Confusion matrix and ROC curve for D_test and I_test**.

**Figure 10.**
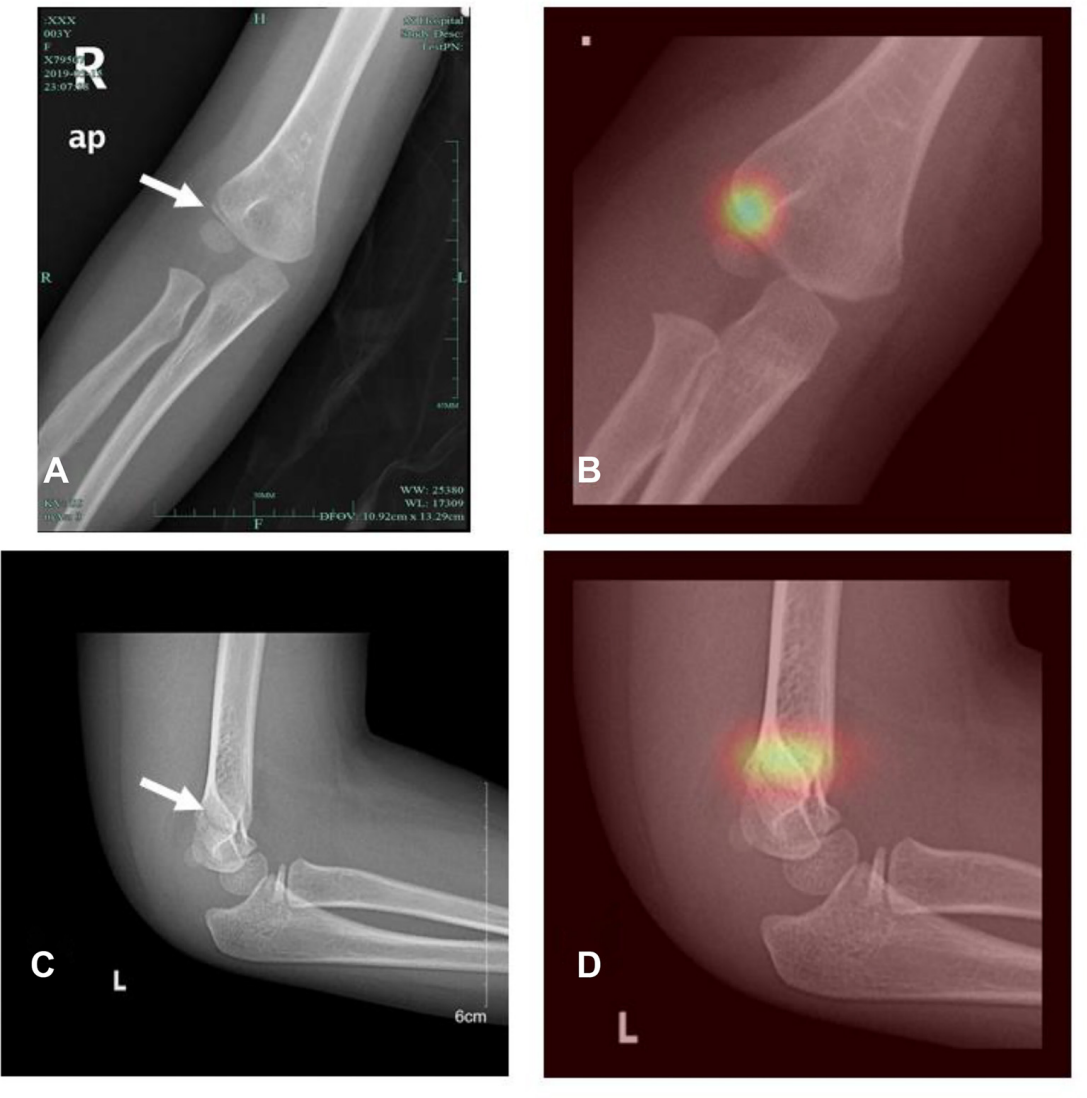
**Heatmap-assisted image identification of a supracondylar fracture.** (A) HZCH elbow radiograph with a subtle supracondylar fracture (arrow); (B) Image generated after detection model; (C) HZCH (2023) radiograph of an elbow fracture (arrow); (D) Heatmap-assisted image. HZCH: Hangzhou Children’s Hospital.

(2) *Results of the repair model*: The performance of the repair model improved substantially with the incorporation of bone edge and texture information. This additional data enabled the model to reconstruct bone structures more effectively. As shown in Figure 8, the model excelled in processing images with clearly defined bone structures, particularly those featuring well-defined bone edges and epiphyseal lines. These results indicate that the model can reliably reconstruct normal anatomical structures, even when bone regions are unclear or only partially visible. The challenge becomes more complex, however, in the presence of fractures. Fracture lines often resemble epiphyseal lines, making accurate fracture detection more difficult. To address this, the repair model focuses on reconstructing the epiphysis, a critical feature in distinguishing fractures from normal anatomical structures. The model can rebuild the normal bone shape in blurred or damaged regions while using its self-residual mechanism to highlight fracture areas. This capability aids in identifying even subtle deviations from normal anatomy. The repair model makes a significant contribution to fracture detection. By reconstructing missing or unclear bone segments and emphasizing fracture regions, it greatly enhances detection accuracy. In the following sections, we provide a more detailed analysis of the model’s impact on fracture detection, including its sensitivity to various fracture types and its potential for clinical application.

(3) *Results of the detection model*: During training, the detection model utilized both images and repaired regions, with sample labels derived from a preprocessed 2D array. The experimental results demonstrate that the MPR model excels in detecting subtle supracondylar fractures. To assess the model’s robustness, we analyzed 200 pediatric elbow X-ray images collected in 2023 from HZCH, all of which contained detailed supracondylar fractures. The confusion matrices for the test and validation sets, along with the ROC curve and AUC values, are displayed in Figure 9. Key evaluation metrics, including sensitivity, specificity, F1 score, and accuracy, are summarized in Table 2. On the test set, the model achieved an accuracy of 94.2%, with a sensitivity of 96.8%, specificity of 91.5%, and an F1 score of 0.943. For the independent validation dataset, the model’s performance was slightly lower but still robust, with an accuracy of 90.5%, sensitivity of 89%, specificity of 92%, and an F1 score of 0.906. These results underscore the model’s strong generalization capability and its effectiveness in managing image variations across different sources. The model outputs a 2D array used for fracture localization. As illustrated in Figure 5, each value in the array corresponds to a specific region in the image. After applying the sigmoid function, the fracture probability for each region is calculated. The resulting heatmap (Figure 10) visually highlights the fracture location. It is important to note that the final image quality may vary due to differences in imaging devices across hospitals.

**Table 2:** Model performance results for D_test and I_test

	**SEN**	**SPE**	**ACC**	**F1**
D_test	0.968	0.915	0.942	0.943
I_test	0.89	0.92	0.905	0.906

(4) *Comparison with doctors*: To evaluate the practical effectiveness and clinical applicability of our model, we compared its performance to the evaluations of two experienced doctors. This comparison is crucial, as the model’s ability to match or surpass human expert judgment serves as a critical benchmark for its reliability and potential in real-world applications. The observer study was conducted exclusively on the I_test dataset, which was independently reviewed by two experienced doctors from HZCH: Y.L. (Pediatric Radiologist) and J.Z.W. (Emergency Physician). The doctors were provided with the original X-ray images but were blinded to clinical information to prevent bias. Both doctors assessed the presence of fractures based solely on the image features and their professional expertise. The evaluation results are summarized in Table 3. The emergency physician achieved an ACC of 0.81 on the I_test dataset, which was notably lower than the performance of our proposed model. This relatively lower accuracy may be attributed to the inherent difficulty of detecting subtle supracondylar fractures. The complex bony anatomy of the elbow can obscure such fractures, making them harder to identify, particularly when they are minor. Additionally, since the emergency physician specializes in acute care rather than radiology, they may have less experience in detecting subtle fractures, potentially leading to occasional oversights. Conversely, the pediatric radiologist performed exceptionally well, achieving significantly higher accuracy compared to the emergency physician. When we compared the model’s results with those of the pediatric radiologist, we found that the model’s accuracy was slightly lower. However, the difference was minimal, underscoring the model’s strong diagnostic capabilities. While the model has demonstrated consistent and reliable performance, these findings also suggest that further refinements could help it match or even surpass the accuracy of expert pediatric radiologists.

**Table 3:** Results from the two physicians in the I_test set

	**SEN**	**SPE**	**ACC**	**F1**
Emergency physician	0.62	0.42	0.81	0.62
Children’s radiologist	0.92	0.942	0.93	0.929

(5) *Comparison with existing algorithms*: To evaluate the performance of our model, we compared it against several well-established fracture detection algorithms, including the ResNet-based DL model [56], the YOLOv8-based model [57], and the recently proposed Transformer-based ViT model [58]. For a fair comparison, all algorithms were trained on the same training dataset (Detection dataset) and validated using the I_test dataset. We assessed their performance using ACC, sensitivity, specificity, and F1 score, with the results summarized in Table 4. As shown in Table 4, our proposed MPR method outperforms the other three algorithms across all evaluated metrics. Specifically, our model achieves a sensitivity of 0.89, a specificity of 0.92, an accuracy of 0.905, and an F1 score of 0.906. When compared to the second-best performer, YOLOv8, our model demonstrates a 0.01 higher sensitivity and a 0.051 higher F1 score, highlighting its superior ability to detect subtle fractures and manage complex scenarios. While the ViT model shows slightly lower overall performance than YOLOv8, it achieves a specificity of 0.90, suggesting a relative strength in minimizing false positives. In contrast, the ResNet-based model underperforms across all metrics, with a sensitivity of just 0.82, which limits its utility for fracture detection tasks. This performance gap is primarily attributed to the impact of limited labeled data on model training, further underscoring the adaptability of our algorithm in addressing challenges posed by scarce labeled datasets.

**Table 4:** Performance comparison of different algorithms on I_test dataset

	**SEN**	**SPE**	**ACC**	**F1**
ResNet-based	0.82	0.84	0.785	0.790
YOLOv8-based	0.88	0.85	0.860	0.855
ViT-based	0.84	0.90	0.825	0.802
Ours	**0.89**	**0.92**	**0.905**	**0.906**

### Ablation study

To assess the effectiveness of each component in enhancing the model’s performance, we conducted a series of ablation experiments. These experiments were designed to evaluate how different types of auxiliary information and modifications affect the accuracy and reliability of the repair and detection models. The experimental results are summarized in Tables 3 and 4. We first analyzed the contribution of auxiliary information to the performance of the repair model. Specifically, we investigated the impact of edge and texture features derived from the Canny and HED methods. As shown in Table 5, the use of these edge detection methods resulted in moderate improvements in repair performance. However, when edge or texture information was applied independently, the performance gains were limited. This finding indicates that while these features positively influence the model, they fail to fully unlock its potential when used in isolation. In contrast, combining HED with the Fourier transform produced a significant boost in the repair model’s performance. This integration allowed the model to capture both structural and frequency-domain information more effectively, thereby maximizing its repair capabilities. These results highlight that the joint application of edge and texture features, alongside Fourier frequency-domain information, offers a more comprehensive understanding of the image, leading to substantial improvements in repair accuracy.

**Table 5:** Ablation studies with different auxiliary information

**Canny**	**HED**	**Fourier**	**SSIM*↑***	**PSNR*↑***	**FID*↓***
✓			0.936	27.92	13.19
	✓		0.946	29.13	10.92
	✓	✓	**0.951**	**30.34**	**9.53**

Next, we evaluate the contributions of the repair module and the optimized loss function (W-BCE) to the crack detection model. The repair module is designed to extract residual information from repaired regions and pass it to the detection model, enhancing crack detection accuracy. Additionally, we analyze the role of the W-BCE loss function, which improves the learning process in the detection task through weighted cross-entropy. The results in Table 6 highlight a significant performance difference between the baseline model using BCE loss and the model incorporating W-BCE. Specifically, the baseline model achieves a SEN of 0.634, whereas the W-BCE model demonstrates a marked improvement, with sensitivity rising to 0.757. This increase indicates that W-BCE effectively directs the network to focus on critical regions of the image, thereby enhancing crack detection accuracy. The substantial performance gap underscores the importance of weighted loss functions in addressing class imbalance issues. In addition, incorporating the repair module improves sensitivity even further, from 0.634 to 0.76. This confirms that the repair module helps the model learn detailed feature information, such as texture and complete edge details, which are crucial for accurate crack detection. These results suggest that the repair module enables the network to capture richer feature representations, leading to improved overall performance. The ablation experiments clearly demonstrate the effectiveness of both the repair module and the W-BCE loss function. The repair module supplies essential supplementary information, enabling the network to learn comprehensive feature representations from both the original and repaired regions. Meanwhile, the W-BCE loss function significantly enhances crack detection by addressing class imbalance and concentrating the learning on the most relevant features. Together, these components deliver the best performance, as evidenced by the substantial improvements in sensitivity and detection accuracy across various evaluation metrics.

**Table 6:** Ablation studies with different parts of the detection model

**BCE**	**Repair area**	**W-BCE**	**SEN**	**SPE**	**ACC**	**F1**
✓			0.634	0.968	0.800	0.750
		✓	0.757	0.989	0.870	0.850
	✓		0.76	1.00	0.880	0.860
	✓	✓	**0.968**	**0.915**	**0.942**	**0.943**

W-BCE: Weighted binary cross-entropy.

## Discussion

We propose a method for detecting subtle pediatric supracondylar fractures using GANs and a multi-scale CNN. To develop the MPR repair model, we address two main challenges: detecting supracondylar fractures in children and the limited availability of labeled datasets. Our approach leverages healthy elbow images for training the repair model and a small, annotated dataset for training the fracture detection and localization module. The fracture detection process begins with cropping the input image using a localization model, ensuring the elbow region is centered with the supracondylar fracture area highlighted. The cropped image is then masked with a small solid region, which incorporates a reconstructed encoder and generator. For normal images, the reconstruction closely resembles the original. In contrast, for abnormal images, the reconstruction aligns with the normal image except for deviations in the fracture area. The reconstructed regions and the original image are subsequently fed into the multi-scale CNN for fracture localization. Unlike traditional methods that rely solely on the original image features or latent space features for classification and localization, our approach uses GANs to learn the distribution of normal data. This enables enhanced feature extraction in abnormal regions, facilitating precise fracture localization through targeted regional repair. By focusing on key differences between normal and abnormal areas, our method significantly improves the accuracy of fracture localization.

We explored the impact of different edge and texture information on image reconstruction. Due to the complex surface structure of pediatric elbows, the Canny edge detector was unable to capture complete edge information. However, the HED and Fourier transform methods proved effective in extracting both edge and texture details, enabling the repair model to generate more realistic images. By training the detection model with the reconstructed regions, we significantly enhanced its ability to recognize and locate fractures. Additionally, we incorporated the W-BCE loss function into the detection model, which optimized fracture detection and localization. Compared to conventional cross-entropy loss, W-BCE accelerated model convergence by placing greater emphasis on fracture locations during training, thereby improving sensitivity and specificity. To validate the effectiveness of our method, we compared the results from our model with those of ED physicians and pediatric radiologists on an independent test set. Our model achieved an accuracy of 90.5%, significantly outperforming the ED physicians (accuracy: 81%) but slightly underperforming the pediatric radiologists (accuracy: 93%). The model’s processing speed (1.1 s per image) further underscores its advantages, particularly in high-throughput clinical settings, where it can significantly improve work efficiency. Additionally, this rapid processing offers potential benefits in emergency situations. For example, in fast-paced ED environments, the model can provide preliminary results on fracture type identification within a very short time, aiding in subsequent treatment decisions. This efficient processing is crucial for clinical decision-making, especially in high-pressure environments with a large patient volume, as it reduces physicians’ workloads and enhances diagnostic efficiency. Despite the model’s strong performance, some challenges remain. Firstly, the model’s accuracy could be influenced by the quality and diversity of the training data. Expanding the training dataset in the future, particularly by incorporating clinical data from a broader range of hospitals and regions, will be vital for improving the model’s generalization and robustness. Secondly, while the current model accurately detects fractures, there is room for improvement in certain complex cases, particularly when image quality is poor or fractures are subtle. Future work will focus on enhancing the model’s adaptability to these challenges, further refining its accuracy and generalization capabilities.

## Conclusion

This study introduces a DL method for detecting pediatric supracondylar fractures using GANs and a multi-scale CNN. The approach effectively tackles major challenges in fracture detection, such as the limited availability of labeled data and the subtle presentation of pediatric fractures, by employing advanced image reconstruction and localization techniques. Tested on an independent dataset, the model achieved 90.5% accuracy, with 89% sensitivity, 92% specificity, and an F1 score of 0.906. These metrics surpass the diagnostic performance of emergency medicine physicians and closely align with that of pediatric radiologists. With its high accuracy and rapid processing speed, this model has the potential to significantly improve diagnostic efficiency, particularly in high-throughput settings such as emergency departments.

## Data Availability

The datasets used and analyzed during the current study are available from the corresponding author on reasonable request.

## References

[1] Schmid A, Lois AM, Metz C, Grunz JP, Veldhoen S (2022 Aug). Not all that looks fractured is broken—multipartite humeral epicondyles in children. Eur Radiol.

[2] Martínez JA, Almero LP, de Anda RC, Botaya EG, Montolio MG, Rey MM (2019). Estudio epidemiológico sobre fracturas supracondíleas de húmero distal en pacientes pediátricos. Rev Esp Cir Ortopédica Traumatol.

[3] Abzug JM, Herman MJ (2012). Management of supracondylar humerus fractures in children: current concepts. J Am Acad Orthop Surg.

[4] Bagaria R, Wadhwani S, Wadhwani AK (2021). Bone fractures detection using support vector machine and error backpropagation neural network. Optik.

[5] Cao Y, Wang H, Moradi M, Prasanna P, Syeda-Mahmood TF.

[6] Sahin ME (2023). Image processing and machine learning-based bone fracture detection and classification using X-ray images. Int J Imag Syst Technol.

[7] Oka K, Shiode R, Yoshii Y, Tanaka H, Iwahashi T, Murase T (2021). Artificial intelligence to diagnosis distal radius fracture using biplane plain X-rays. J Ortho Surg Res.

[8] Mawatari T, Hayashida Y, Katsuragawa S, Yoshimatsu Y, Hamamura T, Anai K (2020). The effect of deep convolutional neural networks on radiologists’ performance in the detection of hip fractures on digital pelvic radiographs. Eur J Radiol.

[9] Guan B, Yao J, Wang S, Zhang G, Zhang Y, Wang X (2022 Feb). Automatic detection and localization of thighbone fractures in X-ray based on improved deep learning method. Comput Vis Image Underst.

[10] Selvaraju RR, Cogswell M, Das A, Vedantam R, Parikh D, Batra D (2020 Feb). Grad-CAM: visual explanations from deep networks via gradient-based localization. Int J Comput Vis.

[11] Yamada Y, Maki S, Kishida S, Nagai H, Arima J, Yamakawa N (2020 Nov). Automated classification of hip fractures using deep convolutional neural networks with orthopedic surgeon-level accuracy: ensemble decision-making with antero-posterior and lateral radiographs. Acta Orthop.

[12] Guan B, Yao J, Zhang G, Wang X (2019 Jul). Thigh fracture detection using deep learning method based on new dilated convolutional feature pyramid network. Pattern Recognit Lett.

[13] Blüthgen C, Becker AS, de Martini IV, Meier A, Martini K, Frauenfelder T (2020 May). Detection and localization of distal radius fractures: deep learning system versus radiologists. Eur J Radiol.

[14] Cha Y, Kim JT, Park CH, Kim JW, Lee SY, Yoo JI (2022). Artificial intelligence and machine learning on diagnosis and classification of hip fracture: systematic review. J Ortho Surg Res.

[15] Dupuis M, Delbos L, Veil R, Adamsbaum C (2022 Mar). External validation of a commercially available deep learning algorithm for fracture detection in children. Diagn Interv Imag.

[16] Crim J (2024). Bone radiographs: sometimes overlooked, often difficult to read, and still important. Skeletal Radiol.

[17] Umadevi N, Geethalakshmi SN.

[18] Lum VL, Leow WK, Chen Y, Howe TS, Png MA.

[19] Al-Ayyoub M, Al-Zghool D (2013 Aug). Determining the type of long bone fractures in x-ray images. WSEAS Trans Inf Sci Appl.

[20] Cao Y, Wang H, Moradi M, Prasanna P, Syeda-Mahmood TF.

[21] Myint WW, Tun KS, Tun HM, Myint H (2018). Analysis on leg bone fracture detection and classification using X-ray images. Mach Learn Res.

[22] Joshi D, Singh TP (2020 Aug). A survey of fracture detection techniques in bone X-ray images. Artif Intell Rev.

[23] Hendrix N, Scholten E, Vernhout B, Bruijnen S, Maresch B, de Jong M (2021 Jul). Development and validation of a convolutional neural network for automated detection of scaphoid fractures on conventional radiographs. Radiol Artif Intell.

[24] Rayan JC, Reddy N, Kan JH, Zhang W, Annapragada A (2019 Jan). Binomial classification of pediatric elbow fractures using a deep learning multiview approach emulating radiologist decision making. Radiol Artif Intell.

[25] Tanzi L, Vezzetti E, Moreno R, Aprato A, Audisio A, Massè A (2020 Dec). Hierarchical fracture classification of proximal femur X-Ray images using a multistage deep learning approach. Eur J Radiol.

[26] Guan B, Zhang G, Yao J, Wang X, Wang M (2020 Jan). Arm fracture detection in X-rays based on improved deep convolutional neural network. Comput Electr Eng.

[27] Naguib SM, Hamza HM, Hosny KM, Saleh MK, Kassem MA (2023). Classification of cervical spine fracture and dislocation using refined pre-trained deep model and saliency map. Diagnostics.

[28] Naguib SM, Saleh MK, Hamza HM, Hosny KM, Kassem MA (2024). A new superfluity deep learning model for detecting knee osteoporosis and osteopenia in X-ray images. Sci Rep.

[29] Naguib SM, Kassem MA, Hamza HM, Fouda MM, Saleh MK, Hosny KM (2024). Automated system for classifying uni-bicompartmental knee osteoarthritis by using redefined residual learning with convolutional neural network. Heliyon.

[30] Kassem MA, Naguib SM, Hamza HM, Fouda MM, Saleh MK, Hosny KM (2023). Explainable transfer learning-based deep learning model for pelvis fracture detection. Int J Intell Syst.

[31] Tanzi L, Audisio A, Cirrincione G, Aprato A, Vezzetti E (2022). Vision transformer for femur fracture classification. Injury.

[32] Sarmadi A, Razavi ZS, van Wijnen AJ, Soltani M (2024). Comparative analysis of vision transformers and convolutional neural networks in osteoporosis detection from X-ray images. Sci Rep.

[33] Nejad RB, Komijani AH, Najafi E (2023). Intelligent cervical spine fracture detection using deep learning methods. arXiv preprint arXiv:2311.05708.

[34] Zhu Z, Chen Q, Yu L, Wang L, Zhang D, Magnier B.

[35] Liu J, Wang H, Shan X, Zhang L, Cui S, Shi Z (2024). Hybrid transformer convolutional neural network-based radiomics models for osteoporosis screening in routine CT. BMC Med Imag.

[36] Makwane S, Kiran A, Bhardwaj S.

[37] Schlegl T, Seeböck P, Waldstein SM, Schmidt-Erfurth U, Langs G (2017). Unsupervised anomaly detection with generative adversarial networks to guide marker discovery. arXiv Mar.

[38] Zimmerer D, Isensee F, Petersen J, Kohl S, Maier-Hein K (2019). Unsupervised anomaly localization using variational auto-encoders. arXiv Jul.

[39] Schlegl T, Seeböck P, Waldstein SM, Langs G, Schmidt-Erfurth U (2019 May). f-AnoGAN: fast unsupervised anomaly detection with generative adversarial networks. Med Image Anal.

[40] Zhao H, Li Y, He N, Ma K, Fang L, Li H (2021 Jul). Anomaly detection for medical images using self-supervised and translation-consistent features. IEEE Trans Med Imag.

[41] Zhou K, Gao S, Cheng J, Gu Z, Fu H, Tu Z (2020). Sparse-GAN: sparsity-constrained generative adversarial network for anomaly detection in retinal OCT image. arXiv Feb.

[42] Kermany DS, Goldbaum M, Cai W, Valentim CC, Liang H, Baxter SL (2018 Feb). Identifying medical diagnoses and treatable diseases by image-based deep learning. Cell.

[43] Zhu X, Lyu S, Wang X, Zhao Q.

[44] Hoang Q, Nguyen TD, Le T, Phung D.

[45] Nazeri K, Ng E, Joseph T, Qureshi F, Ebrahimi M.

[46] Li J, He F, Zhang L, Du B, Tao D.

[47] Xie S, Tu Z.

[48] Canny J (1986). A computational approach to edge detection. IEEE Trans Pattern Anal Mach Intell.

[49] Chen M, Zhao S, Liu H, Cai D (2020 Apr). Adversarial-learned loss for domain adaptation. Proc AAAI Conf Artif Intell.

[50] Johnson J, Alahi A, Fei-Fei L.

[51] Lin TY, Dollár P, Girshick R, He K, Hariharan B, Belongie S.

[52] Lin TY, Dollár P, Girshick R, He K, Hariharan B, Belongie S.

[53] Bussola N, Marcolini A, Maggio V, Jurman G, Furlanello C.

[54] Tampu IE, Eklund A, Haj-Hosseini N (2022). Inflation of test accuracy due to data leakage in deep learning-based classification of OCT images. Sci Data.

[55] Yagis E, Atnafu SW, García Seco de Herrera A, Marzi C, Scheda R (2021). Effect of data leakage in brain MRI classification using 2D convolutional neural networks. Sci Rep.

[56] Wang H, Ying J, Liu J, Yu T, Huang D (2024). Harnessing ResNet50 and SENet for enhanced ankle fracture identification. BMC Musculoskelet Disord.

[57] Ju RY, Cai W (2023). Fracture detection in pediatric wrist trauma X-ray images using YOLOv8 algorithm. Sci Rep.

[58] Chład P, Ogiela MR (2023). Deep learning and cloud-based computation for cervical spine fracture detection system. Electronics.

